# Bone damage after chemotherapy for lymphoma: a real-world experience

**DOI:** 10.1186/s12891-021-04904-3

**Published:** 2021-12-07

**Authors:** S. Mancuso, Dalila Scaturro, M. Santoro, G. Di Gaetano, F. Vitagliani, V. Falco, S. Siragusa, S. Gonnelli, G. Letizia Mauro

**Affiliations:** 1 Department of Health Promotion, Mother and Child Care, Internal Medicine and Medical Specialties (PROMISE), Hematology Unit, University of Palermo, Palermo, Italy; 2 Department of Surgical, Oncological and Stomatological Disciplines, University of Palermo, Via del vespro 129, 90127 Palermo, Italy; 3 University of Palermo, Palermo, Italy; 4 University of Catania, Catania, Italy; 5 Department of Economics Business and Statistic, University of Palermo, Palermo, Italy; 6 Department of Medical and Surgical Sciences and Neurosciences, Respiratory Diseases and Lung Transplantation, Siena University Hospital, Siena, Italy

**Keywords:** Chemotherapy, Osteoporosis, Lymphoma, Steroids, Bone losses, Osteoclastic

## Abstract

**Background:**

Despite recent improvements in survival due to advances in treatment, the quality of life of patients with lymphoma may be compromised by the long-term complications of chemotherapy and steroid therapy. Among these, a potentially relevant problem is bone loss and the development of fragility fractures.

**Aim:**

To provide further evidence of clinical or subclinical skeletal complications in correlation with biological variables and markers of bone disease in patients with complete response to therapy.

**Method:**

A cross-sectional observational study was conducted on subjects diagnosed with lymphoma with subsequent antineoplastic treatment, disease status after therapy defined as complete response disease for at least a year now. We performed: blood chemistry tests, imaging techniques and screening tools for the assessment of functional status and quality of life (SARC-F and mini-Osteoporosis Quality of Life).

**Results:**

Approximately 50% of patients had osteoporosis, with a prevalence of vertebral fractures of 65.5%. In most patients, we found hypovitaminosis D and high levels of parathyroid hormone (PTH). Furthermore, a statistically significant association was observed between high PTH levels and previous lymphoma treatment. Finally, the Mini-Osteoporosis Quality of life (mini-OQLQ) questionnaire demonstrated a loss of quality of life as a consequence of the change in bone status.

**Conclusions:**

Patient treatment design for personalized chemotherapy would be desirable to reduce late effects on bone. Also, early prevention programs need to be applied before starting treatment. The most benefited subpopulations could be not only elderly but also young patients.

## Introduction

Despite recent improvements in survival because of advances in treatment, quality of life of patients with lymphoma may be compromised by long-term complications of chemo and steroid therapy [1]. A potentially relevant issue for lymphoma survivors is bone loss - osteopenia and osteoporosis- as consequence of the treatment. Osteoporosis can lead to development of fragility fractures, a major cause of morbidity associated with considerable mortality [2].

Some studies have evaluated the occurrence and the impact of bone loss in adult patients with hematological neoplasms including lymphomas [3]. Baseline testing of bone mineral density (BMD) reveals osteopenia or osteoporosis in the majority of Non Hodgkin lymphoma (NHL) patient s[4].Patients with lymphoma have significant bone loss compared to the normal general populatio n[5], because it is known that the therapy of lymphoma with high-dose glucocorticoids and alkylating agents may result in premature bone loss, increasing the risk of vertebral and hip fractures. It is also observed that low BMD seen at diagnosis may worsen after lymphoma therapy [6, 7].

Glucocorticoids increase bone resorption and reduce bone formation by inhibiting the formation of osteoblast precursors and inducing apoptosis of mature osteocytes. They also determine a reduction in intestinal absorption of calcium and muscle mass. Alkylating agents, in addition to their important cardiotoxic effect [8], also negatively influence bone metabolism through their genotoxic action which determines early menopause in these patients, especially of female sex [9].

The effects on the bone of individual drugs and combined therapeutic regimes are complex, and the lack of clear knowledge about molecular pathways and regulatory events does not make it easy to implement effective prevention strategies in patients with lymphoma [10].

To date, very few studies have described functional status of bone with comprehensive analysis of clinical features, biochemical parameters and imaging studies in previously treated patients for lymphoma.

Unlike hormone-sensitive tumors undergoing hormone replacement therapy, such as breast cancer and prostate cancer, where there is now unanimous agreement on the role and effectiveness of primary preventive therapy on bone health, in the case of lymphomas, most patients treated with chemotherapy do not receive prior treatment of osteoporosis, although some studies have shown a clear loss of bone mass in these patients, especially in the first two years after treatmen t[9–11].

A critical role in bone mineral metabolism is played by PTH and vitamin D. They form a tightly controlled feedback loop, as PTH is one of the main stimulators of vitamin D synthesis in the kidney, while vitamin D exerts negative feedback on the PTH secretion. PTH is the main physiological regulator of serum calcium concentration. Through its effects on the intestines, kidneys, and bones it keeps serum calcium within a narrow range. Conversely, vitamin D has a stimulating effect on both calcium and phosphate homeostasis, playing a key role in providing adequate mineral for normal bone formation [12].High levels of PTH and/or low levels of Vitamin D can promote the onset of osteoporosis. In patients with lymphoma, a condition of Vitamin D deficiency/insufficiency is often identified, correlating with a worse outcome of the disease. Furthermore, vitamin D also appears to play an important role in malignant hematological cells. In the latter, supplementing with vitamin D promotes apoptosis and inhibits its proliferation. Although the dosage of vitamin D required to achieve these effects may induce hypercalcemia in humans, analogs have been developed that can avoid this side effect [13, 14].

This real-world study was conducted to provide further evidence of clinical or subclinical skeletal complications, possibly correlating with major bone disease markers (such as Vitamin D and PTH), and ultimately to assess the quality of life in relation to sarcopenia in patients with complete response lymphoma to treatment.

## Patients and methods

### Study design and population

This is an observational, cross-sectional study conducted at Policlinico “PaoloGiaccone” of Palermo. within an interdisciplinary setting involving Hematology Unit and Physiatric Unit. The patients were recruited from March 2018 to July 2020. The study was conducted in accordance with the Declaration of Helsinki and with the approval from the Ethical Committee, n. 02/2018. All participants provided written informed consent before study entry.

Inclusion criteria comprised: age between 16 and 85 years, previous diagnosis of lymphoma with subsequent antineoplastic treatment, disease status after therapy defined as complete response for at least a year [9]. Exclusion criteria were as follows: patients with progressive disease following first-line chemotherapy; patients treated with more than 1 line of chemotherapy; any previous o current treatment for osteoporosis; other concomitant malignancies both with and without metastasis; concurrent systemic inflammatory rheumatic disease; history of metabolic bone disease; renal insufficiency stage 4 and 5; any medical comorbidity that may cause osteoporosis and/or other variables alterations; physical disabilities that may prevent the patient to fully understand and adhere to study procedures; patients with previous known vertebral fractures for non-osteoporotic reasons; patients not able to understand and give informed consent.

### Assessments

Baseline data regarding demographic and clinical information was collected from clinical charts. Variables collected from this source were: age, gender, body mass index (BMI), type of lymphoma, histotype of lymphoma, treatment protocol received, and comorbidities. At the time of the physiatric consultation, other variables were evaluated for the evaluation of bone metabolism, such as blood levels of Vitamin D and PTH. Furthermore, BMD, T-score and Z-score of the femoral and lumbar spine were also evaluated using the densitometric examination, and through thoracolumbar radiographic scans with morphometric count according to the Genant criteria, the presence of vertebral fragility fractures was assessed.The following evaluation questionnaires were also administered to all patients: SARC-F questionnaire, for the screening of sarcopenia; and Mini-OQLQ to assess the quality of life in relation to osteoporosis.

For the measurement of vitamin D levels, we used the following reference cut-off:20 ng/ml deficiency, 20–30 ng/ml insufficiency, and normal 30–100 ng/m l[15].The reference cut-offs used for the measurement of parathormone levels were:70 pg/Ml normal values and 70 pg/ml high values (hyperparathyroidism )[16].

Dual-energy x-ray absorptiometry (DEXA) scans of the femoral neck and lumbar spine were performed to detected BMD. According to the World Health Organization criteria, osteopenia is defined as a T score between − 1 > − 2.5, and osteoporosis is defined as a T score > − 2.5 or les s[17].

Genant morphometric counting (or semi-quantitative criteria) is a radiological method used to identify the presence of vertebral fractures and applies to vertebrae T4 to L4. According to these criteria, a normal vertebra is defined as grade 0. A grade 1 deformity is defined by a reduction of 20–25% of the anterior, middle, and/or posterior vertebral height and 10–20% of the area. A grade 2 deformity is defined by a reduction of 26–40% in any height and 20–40% in the area. Finally, a grade 3 deformity is defined by a reduction of > 40% of any height and are a[18].

The SARC-F questionnaire (five-voice questionnaire) is a screening tool for sarcopeni a[19, 20].SARC-F includes five components: strength, assistance walking, rise from a chair, climb stairs, and falls. Scale scores range from 0 to 10 (i.e. 0–2 points for each component; 0 = *best* to 10 = *worst*).

The mini-OQL Q[21]includes two items in each of five domains (symptoms, physical function, activities of daily living, emotional function, leisure activities). For each of the 10 questions a mark between 1 and 7 is assigned: score 1 corresponds to the worst possible function (extreme difficulty, permanent fear and extreme anxiety), instead score 7 is associated with the better function possible (absence of difficulty, of fear and anxiety). The total score of the questionnaire can vary from minimum of 10 to maximum of 70. The data collected were analyzed by calculating mean, median and mode values of total score. Finally, a possible correlation has been evaluated between osteoporosis secondary to lymphoma and some of the main markers of bone metabolism such as PTH and Vitamin D, also in consideration of other demographic variables (age, sex) and clinical (type of treatment, comorbidity, sarcopenia, and quality of life).

### Statistical analysis

All the information collected from patients enrolled were entered into an electronic database created by Excel 16.0 software.

All quantitative values were expressed as mean ± SD or median with interquartiles. The Kolmogorov–Smirnov test was used to verify thenormality of the distribution of the study variables. Absolute and relative frequencies were obtainedto the data collected: age, sex, BMI, Vit D, PTH, femoral BMD and vertebral BMD, type of lymphoma, comorbility, number of vertebral fractures, DEXA femoral, SARC-F and Mini-OQoL.

The sample was stratified and analysed trough a univariate analysis by Vitamin D and Parathormone levels.

The differences in the categorical variables for levels of Vitamin D and Parathormone were analysed using Mann-Whitney test for the quantitative variables and Fisher exact test for categorical variables. Since the sample size is low, we use a non-parametrical test on the medians and an exact test to evaluate the associations. We also used the Pearson correlation coefficient to evaluate the linear correlation among some quantitative variables. The level of significance chosen for the univariate analysis was 0.05 (two tailed). All the data were analysed using the statistical software R (R Core Team, 2021).

## Results

A total of 29 patients previously treated with lymphotoxictherapy (chemotherapy, radiotherapy, steroid) for lymphoma were evaluated. Overall demographic and clinical features are shown in Table 1.

**Table 1:** Blood chemistry values, BMD findings by DEXA, verterbral fractures, sarcopenia assessment

		N° (%)	Mean (SD)
Sex	*Male*	19 (65.5)	
	*Female*	10 (34.5)	
Age			61.4 ± 16.7
BMI			27.1 ± 4.3
Diagnosis	*Hodgikin Lymphoma*	4 (13.8)	
	*Non-Hodgikin Lymphoma*	25 (86.2)	
Histopathology (NHL)	*DLBCL*	12 (48.0)	
	*FL*	6 (24.0)	
	*MZL*	4 (16.0)	
	*CLL*	1 (4.0)	
	*MycosisFungoides*	1 (4.0)	
	*LGL leukemia*	1 (4.0)	
Comorbidities	*≥ 3*	12 (41.4)	
	*1 or 2*	10 (34.5)	
	*No*	7 (24.1)	
Chemotherapy	*Yes*	29 (100.0)	
	*No*	0 (0.0)	
Radiotherapy	*Yes*	4 (13.8)	
	*No*	25 (86.2)	
Corticosteroid Therapy	*Yes*	17 (58.6)	
	*No*	12 (41.4)	

The patient group included 18 males (62.1%) and 11 females (37.9%). The mean age of the entire study group was 61.4 years (SD 16,7). The average BMI was 27.1 ± 4.3.4 subjects (13.8%)had a diagnosis of Hodgkin Lymphoma (HL), and 25 (86.2%) of NHL. In the 25 patients with NHL, the most common histology was: large B cell lymphoma (DLBCL) (48%), follicular lymphoma (FL) (24%), and marginal zone lymphoma (MZL) (16%).Overall, 12 patients (41.4%) reported 3 or more comorbidities and 10 patients (34.5%) one or two.All recruited patients received chemotherapy treatment, while only 4 (13.8%) also received radiotherapy and 17 patients received corticosteroid therapy.

Table 2 shows the results of the variables analyzed for bone metabolism.The average vitamin D levels in the sample were 21.6 ng/ml (SD 8.8): in 5 patients they were normal (17.2%), while insufficient and deficient respectively in 22 (75.9%) and 2 (6.9%) cases. PTH values were high in 6 patients (20.7%), in the normal range in 20 cases (69%) and low in 3 cases (10.3%) with an average of 43.5 pg/ml (SD 26.8).

**Table 2:** Variables for the evaluation of bone metabolism

		No. (%)	Mean ± SD
Vitamin D (ng/ml)	*Normal (31–100)*	5 (17,2)	21.6 ± 8.8
	*Insufficiency (11–30)*	22 (75,9)	
	*Deficiency (≤10)*	2 (6,9)	
PTH (pg/ml)	*High (> 70)*	6 (20,7)	43.5 ± 26.8
	*Normal (12–70)*	20 (69,0)	
	*Low (< 12)*	3 (10,3)	
BMD Femural (g/cm2)			0.9 ± 0.1
T-Score Femural	*Normal*	15 (55,6)	−0.8 ± 1.2
	*Osteopenia*	10 (37,0)	
	*Osteoporosis*	2 (7,4)	
Z-Score Femural			−0.1 ± 1.1
BMD Lumbar Vertebral			0.9 ± 0.2
T-Score Lumbar Vertebral	*Normal*	14 (50,0)	−1.0 ± 1.5
	*Osteopenia*	7 (25,0)	
	*Osteoporosis*	7 (25,0)	
Z-Score Lumbar Vertebral			−0.2 ± 1.4
Vertebral Fractures	*No*	10 (34,5)	2.1 ± 1.8
	*1 or 2*	10 (34,5)	
	*≥3*	9 (31,0)	
SARC-F	*≥4 (sarcopenic)*	18 (62)	3.8 ± 1.9
	*< 4 (non sarcopenic)*	11 (38)	
Mini OQoL	*Mild*	11 (37,9)	
	*Moderate*	16 (55,2)	54.4 ± 11.1
	*Severe*	2 (6,9)	

Femural DEXA was determined in 27 patients, while the other 2 patients had bilateral hip prostheses, with these results of T-score: normal in 15 cases (55.6%), osteopenia in 10 (37%), osteoporosis in 2 (7.4%). The calculated average T-score was − 0.8 (DS 1.2) and the average Z-score − 0.1 (DS 1.1). Vertebral lumbar DEXA was performed in 28 patients, because one hadhad vertebral stabilization:14 of them (50%) had normal findings, 7 (25%) had osteopenia and 7 (25%) osteoporosis. The average calculated T-score was − 1 8 (DS 1.5) and the average Z-score was − 0.2 (DS 1.4).

As for vertebral fractures, 10 (34.5%) patients had no fractures, but 10 (34.5%) had 1 or 2 fractures and 9 (31%) had 3 or more than one fracture. The average value was 2.1 (DS 1.8).

**Fig. 1:**
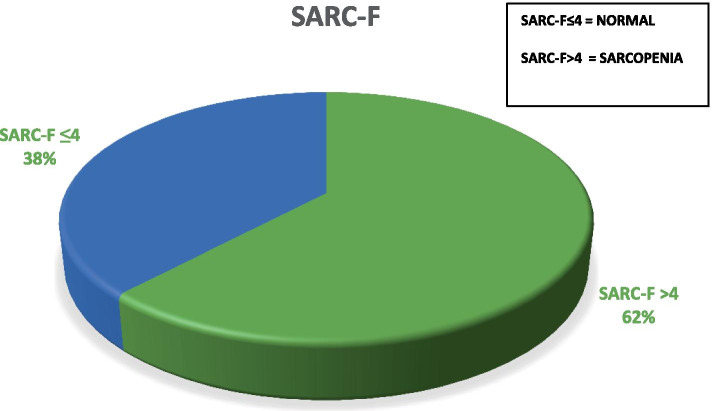
Percentage of patients with SARC-F values > 4 and ≤ 4

The average SARC-F values were 3.8 (DS 1.9). In Fig. 1 shows the distribution of patients with a SARC-F of less than 4 points (38%) compared to those with a score of 4 or more (62%).

**Fig. 2:**
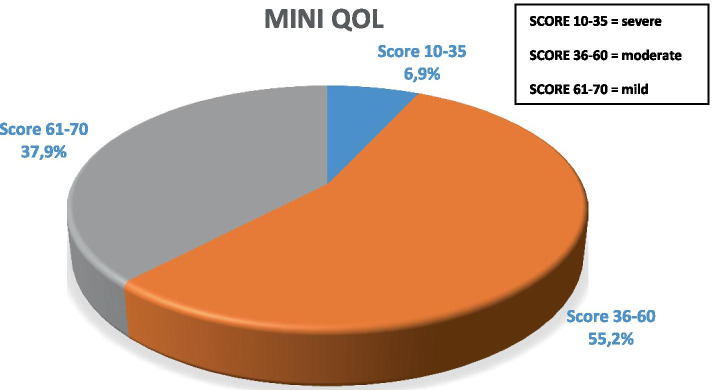
Mini QoL score

MINI-OQoL detected a mean total score of 54.4 ± 11.1 and a median total score of 55 (range 32–70). In Fig. 2, patients were classified by scores obtained at the questionnaire: the 37.9% of the patients was classified with a mild OQoL score (> 60 points), the 55.2% had a moderate score (36–60 points), while the 6.9% had a severe score (< 36 points). So the observed mode is the moderate score (36–60 points).

Table 3 shows the results of the correlation between the blood values of Vitamin D and PTH and the demographic and clinical characteristics of the patients.

**Table 3:** Association of Vitamin D and Parathormone values with socio-demographic and clinical characteristics of the patients

		Vitamin D		*p*-value	Parathormone		*p*-value
		Reference	Insufficiency /Deficiency		High	Reference	
Diagnosis	*Hodgkin lymphoma*	1 (25.0)	2 (75.0)	0.46	0 (0.0)	3 (100.0)	0.56
	*Non-Hodgkin lymphoma*	4 (16.0)	21 (84.0)		6 (23.1)	20 (76.9)	
Age		63.0 (21.0)	66.0 (26.5)	0.49	62.0 (23.5)	77.0 (4.5)	**0.02**
Gender	*Male*	5 (26.3)	14 (73.7)	0.14	5 (26.3)	14 (73.7)	0.63
	*Female*	0 (0.0)	9 (100.0)		1 (10.)	9 (90.0)	
Chemo and steroid therapy	*Yes*	2 (12.5)	14 (87.5)	0.63	6 (37.5)	10 (62.5)	**0.02**
	*No*	3 (23.1)	10 (76.9)		0 (0)	13 (100)	
Comorbidities	*Yes*	4 (18.2)	18 (81.8)	0.99	6 (27.3)	16 (72.7)	0.15
	*No*	1 (14.3)	6 (85.7)		0 (0)	7 (100)	
Vertebral fractures	*Yes*	2 (10.5)	17 (89.5)	0.29	6 (31.6)	13 (68.4)	**0.05**
	*No*	3 (33.3)	6 (66.7)		0 (0.0)	10 (100)	
Femoral DEXA	*Normal*	3 (20.0)	12 (80.0)	0.83	3 (20.0)	12 (80.0)	0.99
	*Osteopenia / Osteoporosis*	1 (9.1)	10 (90.9)		3 (25.0)	9 (75.0)	
Vertebral DEXA	*Normal*	2 (14.3)	12 (85.7)	0.57	3 (21.4)	11 (78.6)	0.93
	*Osteopenia / Osteoporosis*	3 (23.1)	10 (76.9)		3 (21.4)	11 (78.6)	
Vitamin D (ng/ml)	*Normal*	/	/	/	0 (0.0)	5 (100.0)	0.30
	*Insuffiency / Deficiency*				6 (26.1)	17 (73.9)	
SARC-F (patients with 1 or 2 points)	*≥4 (sarcopenic)*	1 (5.9)	16 (94.1)	**0.06**	3 (16.7)	15 (83.3)	0.65
	*< 4 (non sarcopenic)*	4 (36.4)	7 (63.6)		3 (27.3)	8 (72.7)	
Mini-OQoL total score	*10–35*	1 (50,0)	1 (50,0)		2 (100,0)	0 (0,0)	0.14
	*36–60*	2 (12,5)	14 (87,5)	0.63	11 (68,7)	5 (31,3)	
	*61–70*	2 (20,0)	8 (80,0)		10 (90,9)	1 (9,1)	

We show that older patients carry significantly higher levels of PTH (*p* = 0.02). In patients undergoing combined treatment with multiple chemotherapeutic agents and high doses of corticosteroids, a significant association with high levels of PTH (p = 0.02) was observed. Furthermore, a significant association was found between higher PTH levels and vertebral fractures (*p* = 0.05).

Table 3 also shows that SARC-F values greater than or equal to 4 points are almost significantly associated with Vitamin D deficiency/deficiency values (*p* = 0.06) with a Pearson correlation coefficient equal to − 0.29 indicative of a moderate negative linear correlation between the two variables. Finally, through Pearson’s correlation, a weak positive linear correlation was also highlighted between the Mini-OQoL score and the Vitamin D values, with a Pearson index of 0.13.

## Discussion

Our study exploring bone damage after lymphoma treatment adds information on the late effects of cancer therapy in survivors of adult patients. In this interdisciplinary study, we identified subjects with lymphoma in complete remission for at least one year, who underwent a screening program for osteoporosis to evaluate the relationship with predictive markers, the presence of vertebral fractures of unknown fragility, and the effects on muscle mass and the quality of life.

Previous studies conducted on this issue generally focused on patients prior to receive front-line therapy or on pediatric survivors, the latter examined, however, before peak bone mass achievement [22].

In the population under examination, we found hypovitaminosis D in 82.8% of patients and high levels of PTH in 20.7%, conditions known to be associated with an increased risk of osteoporosis [23]..

Vitamin D deficiency is a known problem in many parts of the world, in fact, 1 in 7 people (14%) are thought to have vitamin D insufficiency or deficiency [24]. The role of vitamin D has been extensively studied in cancer patients however the conclusions are sometimes conflicting [25, 26]. Some authors state that low serum levels of vitamin D are associated with a higher risk of developing NHL, which is also confirmed by other studies that conclude that sun exposure favors higher serum levels of vitamin D reducing the risk of developing NHL [25]. Furthermore, it is now known that vitamin D deficiency represents a negative prognostic factor for patients with lymphoma, treated with chemotherapy in particular Rituximab plus cyclophosphamide, doxorubicin, vincristine and prednisone (R-CHOP) [26]*,* in fact, vitamin D supplementation can be useful to improve survival in these patients. The reduced blood concentration of Vit D in cancer patients can be a consequence not only of the antineoplastic treatment but also secondary to a condition of inactivity, isolation, and loss of appetite. In the aforementioned patients, a picture of sarcopenia is often established. In patients with lymphomas, we found that insufficient Vitamin D values were related to sarcopenia. More than half of the patients had a SARC-F questionnaire score at risk for sarcopenia. Sarcopenia combined with osteopenia/osteoporosis increases the chances of fracture [27–29].. The complex relationship between sarcopenia and chemotherapy has been studied in the literature, as they create a vicious circle for musculoskeletal complications [30], assuming that antineoplastic drugs can affect muscle cells and worsen their function. It is now known that chemotherapeutics cause a reduction in muscle mass much faster than that related to normal aging. This is achieved through their myotoxic effects, as they cause mitochondrial dysfunction and oxidative stress, promoting skeletal muscle atrophy. All this is responsible for the high prevalence of asthenia, muscle weakness, and physical disability of cancer patients. This situation contributes to increasing inactivity and reducing the ability to stay outdoors and expose oneself to sunlight, the main source of Vit D production. Chemotherapy-induced sarcopenia is considered to be an unfavorable prognostic factor for survival [30–32]..

This finding suggests that vitamin D dosage may help to identify patients not only at risk of developing fractures but also already suffering from musculoskeletal metabolic alterations that may worsen the quoad valetudinem prognosis of patients with lymphoma. To date, this is the first approach to assess osteoporosis-related health status in lymphoma patients [33–35].

In addition to the above, in our study, we found that worse mini-OQoL scores are related to low vitamin D levels, with a moderate/severe impact on more than half of the population examined. The above supports the observation in real life that supplementation with vitamin D improves the ability to perform activities of daily life. The positive effects of vitamin D on quality of life have been widely reported in the literature. It can be considered an antioxidant agent as it can reduce the oxidative damage of proteins by reducing harmful carbonyl proteins. Furthermore, supplementation of vitamin D seems to induce an improvement in mood, although this effect has not been shown in cancer patients. As a consequence of these actions, vitamin D determines an improvement in muscle function and quality of life [36, 37].

Consistent with what happens in the general elderly population, hyperparathyroidism is also a condition associated with advanced age in subjects with lymphomas. Normally in the elderly population serum, PTH levels are often higher than those in the younger population. All this is associated with an increased risk of osteosarcopenia in the elderly, with poor functional status and an increased prevalence of falls and therefore fractures [38–40].

In our study, we also noted how hyperparathyroidism is also associated with antineoplastic treatment combined with chemotherapy and corticosteroids [41]. This is even more relevant given the statistically significant correlation between vertebral fractures and hyperparathyroidism in the study population. This confirms the contribution of PTH in predicting bone damage in the population studied. However, these observations do not definitively establish the functional importance of high PTH levels in individuals with lymphoma, but knowledge about the healthy population could be translated into patients with lymphoma [42, 43].

Further evidence of the negative impact of antineoplastic treatment on bone was highlighted by the finding of osteopenia and osteoporosis on DEXA in about half of the patients, particularly considering the presence of many young adults in the study group. of greater importance was the finding, in 65.5% of cases, of vertebral fragility fractures, given their implications on morbidity and mortality.

These results are consistent with previous studies, i.e. systemic exposure to steroids at high doses can increase osteoporosis and fracture risk [42, 43]. Corticosteroids, especially when used chronically, lead to severe adverse effects on the musculoskeletal system. At the bone level, they increase the risk of osteoporosis, as they lead to an increase in bone resorption and a reduction in bone formation, by inhibiting osteoblast differentiation and osteocyte apoptosis. At the muscular level, on the other hand, they are responsible for proximal myopathy and muscle atrophy. These combined effects increase the prevalence of falls resulting in an increased risk of bone fractures [42, 43].

In relation to the data obtained, it would be desirable to measure the blood levels of Vitamin D and PTH, the execution of a DEXA, and radiographic examination of the thoracolumbar spine between routine examinations in patients with lymphoma, in order to counteract the negative effects on the bone early.

Limitations of our study include the sample size and selection bias inherent to different lymphoma subtypes with different chemotherapy protocols. Together, these data suggest that simple clinical check in asymptomatic patients is not yet sufficiently sensitive to screen bone loss or fractures after chemotherapy, it is necessary to supplement with humoral assays and imaging techniques.

Thus, upcoming prospective studies with larger case numbers are needed to further validate the best approach for monitoring bone damage in lymphoma patients, before and after chemotherapy.

## Conclusioni

Despite generally favourable outcome, lymphoma patients experience an assortment of late complications.Only recently the term “lymphoma survivorship” was coined and researchers have begun to assess long-term side effects of antineoplastic therapy. In addition to cardiac concerns, infertility and secondary cancers, the musculoskeletal effects have been recognized as one of the most common potential risks [44–46]. Prevention and treatment of the bone damage in lymphoma patients often plays a minor role in clinical practice.

According to the results of our study, performing screening tests for osteoporosis in patients suffering from lymphomas should be recommended and should be included in clinical practice among the routine tests to be performed. Subjects at risk of osteoporosis. Despite bone damage can vary considerably, depending onparticular chemotherapy regimen and age, our current investigation supports a rationale for the hypothesis that lymphoproliferative diseases and chemotherapy are itemsthat have to be taken into consideration for fracture risk stratification.

The study results suggest designing personalized lymphoma therapy and building an accurate follow-up plan, including bone health surveillance in long-term survivors, to identify adverse events or, at least, diagnosing them promptly. Furthermore, it is necessary to apply for prevention programs before starting treatment, even in young patients, being a potentially not considered, but potentially affected subpopulation.

## Abbreviations

PTH: Parathormone
BMD: body mass density
BMI: body mass index
HL: Hodgkin lymphoma
NHL: Non-Hodgking lymphoma
DEXA: Dual energy X-ray absorptiometry
mini-OQLQ: Mini-Osteoporosis quality of life questionnaire
DLBCL: large B cell lymphoma
FL: follicular lymphoma
MZL: marginal zone lymphoma
R-CHOP: Rituximab plus cyclophosphamide, doxorubicin, vincristine and prednisone
